# Two-cohort machine learning approach for predicting the risk of secondary hyperlipidemia in patients with depression

**DOI:** 10.3389/fendo.2026.1769189

**Published:** 2026-05-04

**Authors:** Ziheng Sun, Xuan Sun, Qi Cai, Ke Lei, Qihang Gao, Min Kang, Yun Shen

**Affiliations:** 1 Department of Pathology, Tongling People’s Hospital, Tongling, Anhui, China; 2 Department of Psychiatry, The Third People’s Hospital of Tongling, Tongling, Anhui, China; 3 Department of Hyperbaric Oxygen Therapy, Tongling People’s Hospital, Tongling, Anhui, China

**Keywords:** decision tree, depressive disorders, LASSO regression, machine learning, predictive modeling, secondary hyperlipidemia

## Abstract

**Background:**

Secondary hyperlipidemia is a common and serious complication in patients with clinically diagnosed depressive disorders, yet early screening tools are lacking. This study aims to develop and validate a machine learning-based model to predict the risk of secondary hyperlipidemia in this population.

**Methods:**

We conduct a retrospective study of 627 patients (mean age: 44.5 ± 13.5 years; 51.9% female). LASSO regression was utilized for feature selection, followed by the development of seven predictive models is used. Model performance is evaluated using the Area Under the Curve (AUC), calibration curves, and Decision Curve Analysis (DCA). The SHapley Additive exPlanations (SHAP) method provides individual-level interpretation.

**Results:**

Six core predictors are identified: Body Mass Index (BMI), weekly physical activity, long-term medication, emotion regulation disorder, C-reactive protein (CRP), and Fasting Plasma Glucose (FPG). Among the evaluated models, the Decision Tree model demonstrates the most clinically appropriate and generalizable performance, with an AUC of 0.87 (95% CI: 0.82–0.92) in external validation.

**Conclusion:**

The integration of machine learning with routine clinical data provides a highly accurate and interpretable tool for the early identification of secondary hyperlipidemia in patients with depression. This approach may facilitate personalized preventive interventions and improve long-term metabolic health outcomes in clinical practice.

## Introduction

With the rapid development of modern society, the pressure on individuals has increased significantly, contributing to the rising prevalence of depressive disorders as defined by established diagnostic criteria such as ICD-10. Recent estimates suggest that depression affects over 300 million people worldwide, with an alarming rise in incidence rates, especially in younger populations (1–3). Depression has become a major public health issue as it imposes a heavy burden on individual well-being and economic costs. It is reported that in the United States alone, the annual economic cost amounts to hundreds of millions of dollars (4). The disease not only significantly impairs daily functioning but also contributes to higher morbidity and mortality rates, highlighting the urgent need for more effective interventions and predictive tools (5).

The association between depression and secondary hyperlipidemia is rooted in complex biological and physiological mechanisms. Chronic psychological stress in depressed patients often leads to the overactivation of the hypothalamic-pituitary-adrenal (HPA) axis, resulting in elevated cortisol levels that promote lipolysis and the hepatic synthesis of very-low-density lipoproteins (6, 7). Furthermore, depression is frequently characterized by chronic low-grade systemic inflammation, which impairs lipid metabolism and insulin sensitivity, thereby creating a physiological environment conducive to the development of hyperlipidemia (8, 9). This creates a vicious cycle, as the presence of metabolic disturbances can worsen depressive symptoms, making timely identification and treatment more challenging (10, 11). Consequently, identifying accessible clinical biomarkers that reflect these underlying pathways is essential for early risk stratification. Inflammatory markers such as C-reactive protein (CRP) have emerged as potential indicators of systemic metabolic stress (12, 13), while metabolic parameters like Fasting Plasma Glucose (FPG) and Body Mass Index (BMI) are crucial for assessing the insulin resistance and adiposity that frequently accompany depressive states (14–16).

However, current research in this area faces several limitations. Many existing studies suffer from small sample sizes, low area under the curve (AUC) values, and a failure to integrate these multidimensional biological and clinical markers (17–19). Furthermore, most studies rely on traditional predictive models that often overlook the complex interplay of psychological, metabolic, and inflammatory factors. Moreover, some studies fail to adhere to established consensus definitions for outcome variables, or they are limited by their geographical scope, such as focusing solely on Western populations (20, 21). Given the complex nature of depression and its multifactorial relationship with other health conditions, Machine learning (ML) offers an innovative approach to identify high-risk individuals by processing these heterogeneous clinical data points to facilitate personalized treatment strategies.

This study aims to address these gaps by incorporating a broader range of variables, including the aforementioned metabolic and inflammatory biomarkers, to create a more robust and predictive model for hyperlipidemia in patients with depression. By utilizing a more diverse dataset and focusing on clinically relevant parameters, this research will provide a comprehensive and practical tool for healthcare providers to identify at-risk individuals and improve clinical outcomes.

## Materials and methods

### Study design and population

**Figure 1:**
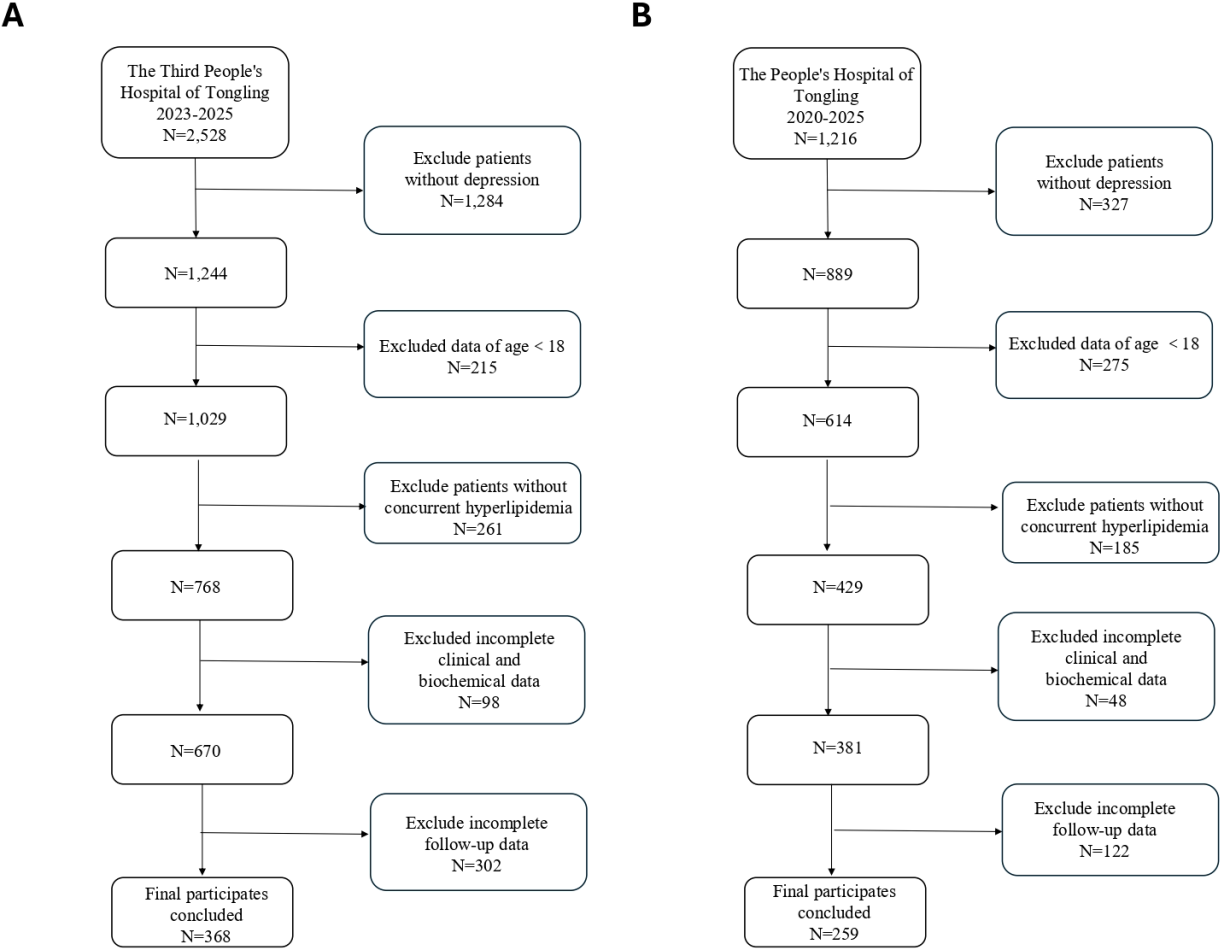
Flowchart of patient inclusion and exclusion. **(A)** Derivation cohort from Tongling Third People’s Hospital (2023–2025). **(B)** External validation cohort from Tongling People’s Hospital (2020–2025). After sequential exclusion of patients without depression, those aged <18 years, those without concurrent hyperlipidemia, and those with incomplete clinical, biochemical, or follow-up data, 368 and 259 participants were included in the derivation and validation cohorts, respectively.

This retrospective study was conducted at two tertiary hospitals in Tongling, Anhui Province, China: Tongling Third People’s Hospital (2023-2025) and Tongling People’s Hospital (2020–2025). Depression was diagnosed according to ICD-10 criteria and further assessed using the Hamilton Rating Scale for Depression (HAMD) to ensure the sample represented a clinically diagnosed depressive disorder population (22–24). After excluding non-depressive patients, minors, and those without sufficient clinical or follow-up data, those aged < 18 years a total of 627 cases (368 from Tongling Third People’s Hospital and 259 from Tongling People’s Hospital) were included in the final analysis (**Figure 1**).

### Inclusion and exclusion criteria

Patients were eligible for inclusion if they met the following criteria: (1) met the ICD-10 diagnostic criteria for depression; (2) were aged 18 years or older; and (3) possessed complete baseline clinical records and laboratory data, including lipid profiles and metabolic indicators. Exclusion criteria included: (1) patients under the age of 18; (2) individuals with documented familial hyperlipidemia or known genetic lipid disorders to ensure the focus remained on secondary hyperlipidemia; (3) presence of severe hepatic or renal dysfunction that could independently interfere with lipid metabolism; (4) pregnancy or breastfeeding. This rigorous selection process ensured that the outcome primarily reflected hyperlipidemia potentially associated with depression and its related clinical or behavioral factors.

### Outcome and predictors

The primary outcome of this study was the presence of secondary hyperlipidemia in patients diagnosed with depression. Hyperlipidemia was defined according to specific clinical lipid thresholds: triglycerides (TG) ≥ 150 mg/dL, total cholesterol (TC) ≥ 200 mg/dL, or low-density lipoprotein cholesterol (LDL-C) ≥ 130 mg/dL, based on laboratory measurements obtained during hospitalization (25).

Participants were screened for a comprehensive set of candidate predictors identified through clinical relevance and existing literature (26–30). These variables included demographic factors (age and sex) and metabolic indicators such as Body Mass Index (BMI), TG, TC, LDL-C, high-density lipoprotein cholesterol (HDL-C), fasting plasma glucose (FPG), and HbA1c. Additionally, inflammatory markers including the neutrophil-to-lymphocyte ratio (NLR) and C-reactive protein (CRP) were recorded, alongside medical history, lifestyle factors (smoking, alcohol consumption, sleep disorders, and physical activity), and psychosocial variables (stress, social support, and emotion regulation disorder).

Long-term medication was defined as the continuous use of prescribed medications over an extended period, reflecting sustained pharmacological treatment; however, specific drug categories (e.g., antidepressants or lipid-lowering agents) were not separately recorded.

### Data preprocessing

Missing data were handled using multiple imputation to preserve sample size and minimize bias from case-wise deletion (31). Five imputations were performed with a fixed random seed for reproducibility. Continuous variables were standardized through Z-score normalization to improve model stability and reduce overfitting.

The dataset from the Third People’s Hospital of Tongling (n = 368) was randomly split into a training set (70%) and an internal validation set (30%) for model development and preliminary evaluation. In addition, an independent external validation cohort was obtained from Tongling People’s Hospital (n = 259), which was not involved in model training. This external cohort was used to assess the generalizability and robustness of the developed models.

Feature selection was performed using LASSO regression with binomial family and L1 regularization (32). Through 10-fold cross-validation and evaluation of 1,000 candidate lambda values, six key predictors—BMI, weekly physical activity, long term medication, emotion regulation disorder, CRP, and FPG—were identified. These features were used in seven machine learning models: logistic regression (LR), decision tree (DT), support vector machine (SVM), artificial neural network (ANN), LightGBM, XGBoost, and random forest (RF). Model performance was assessed using calibration curves, decision curve analysis (DCA), Receiver Operating Characteristic (ROC) curves, and external validation.

### Machine learning algorithms

Seven machine learning algorithms were employed in this study: logistic regression (LR), decision tree (DT), random forest (RF), support vector machine (SVM), artificial neural network (ANN), XGBoost, and LightGBM. Hyperparameter optimization was performed using grid search and five-fold cross-validation.

The decision tree model achieved the best performance with the following parameters: {‘ccp_alpha’: 0.0, ‘max_depth’: 5, ‘max_features’: ‘sqrt’, ‘min_samples_leaf’: 5, ‘min_samples_split’: 10}, effectively controlling model complexity while maintaining predictive power. The random forest model performed optimally with n_estimators = 250 and max_features = ‘sqrt’, ensuring robustness and preventing overfitting. The best XGBoost model was configured with {‘colsample_bytree’: 0.6, ‘learning_rate’: 0.01, ‘max_depth’: 7, ‘n_estimators’: 100, ‘subsample’: 0.6, balancing accuracy and computational efficiency. LightGBM showed superior performance with {‘colsample_bytree’: 0.6, ‘learning_rate’: 0.01, ‘max_depth’: 3, ‘n_estimators’: 200, ‘num_leaves’: 20, ‘subsample’: 0.6}, optimizing both model accuracy and training speed. The SVM model was fine-tuned with {‘C’: 100, ‘class_weight’: ‘balanced’, ‘coef0’: 1, ‘degree’: 4, ‘gamma’: 0.001, ‘kernel’: ‘poly’, ‘shrinking’: True, ‘tol’: 0.0001}, providing a robust classification model. Lastly, the ANN model yielded optimal results with {‘activation’: ‘tanh’, ‘alpha’: 0.0001, ‘hidden_layer_sizes’: (50), ‘max_iter’: 500}, facilitating effective learning in a complex feature space.

### Model evaluation

To assess model performance, several evaluation metrics were used, including ROC curves, Area Under the Curve (AUC), calibration curves, and decision curve analysis (DCA). ROC curves were plotted to evaluate the model’s discriminatory ability, showing the trade-off between sensitivity and specificity at different threshold values. The AUC (Area Under the Curve) quantifies this trade-off, with a higher AUC indicating better overall performance in distinguishing between the positive and negative outcomes. Calibration curves were used to assess the agreement between predicted probabilities and actual outcomes, reflecting how well the model’s predictions align with real-world results. Decision curve analysis (DCA) was performed to evaluate the clinical utility of the models by calculating the net benefit at different threshold probabilities, thus guiding the selection of optimal decision thresholds for clinical applications.

External validation was conducted using an independent dataset to evaluate the generalizability of the model. This step ensured that the model’s performance was not limited to the training or validation datasets but held across different populations and settings, further confirming its clinical applicability.

### SHAP interpretability analysis

To enhance the interpretability of the machine learning model, SHAP (Shapley Additive Explanations) values were calculated to quantify the contribution of each predictor to the model output. SHAP was based on Shapley values from cooperative game theory and estimated the contribution of each feature to the final prediction by considering different feature combinations.

For each prediction, SHAP values indicated the direction and magnitude of a feature’s effect on the outcome relative to the baseline prediction. Positive SHAP values were associated with a higher predicted probability of the outcome, whereas negative values were associated with a lower predicted probability.

In this study, SHAP values were calculated using the TreeSHAP algorithm for tree-based models. SHAP summary plots were used to visualize the overall importance and effect direction of each feature in predicting secondary hyperlipidemia.

### Statistical analysis

Statistical analyses and model development were performed using R v4.5.1 and Python v3.10. To achieve the study objectives, the analytical process was divided into four stages. First, for data preprocessing, missing values were addressed using multiple imputation; continuous variables were expressed as mean ± SD or median (IQR) and compared via Student’s t-test or Wilcoxon rank-sum test, while categorical variables were analyzed using χ^2^ or Fisher’s exact test. Second, to identify the most relevant predictors and minimize overfitting, LASSO regression with 10-fold cross-validation was employed for feature selection. Third, the identified features were integrated into seven machine learning algorithms (LR, DT, SVM, ANN, LightGBM, XGBoost, and RF) to develop the predictive framework. Fourth, model performance and clinical utility were rigorously evaluated using Receiver Operating Characteristic (ROC) curves (expressed as Area Under the Curve, AUC), calibration curves (Brier score), and Decision Curve Analysis (DCA). Finally, the SHAP method was applied to provide individual-level model interpretation. Statistical significance was defined as a two-tailed P-value of <0.05.

## Result

### Patient characteristics

The baseline characteristics of participants from the Tongling Third People’s Hospital (**Table 1**) and Tongling People’s Hospital (external validation cohort, **Table 2**) are summarized. Significant differences were observed between the two cohorts in several variables. At the Third People’s Hospital cohort, the prevalence of obesity was notably higher in the hyperlipidemia group (68%) compared to the non-hyperlipidemia group (27.57%, P < 0.001). Similarly, a significant difference in weekly physical activity was found, with 100% of the hyperlipidemia group reporting <150 minutes of weekly physical activity (P < 0.001). Long-term medication uses also showed a significant disparity, with 77.60% of the hyperlipidemia group using long-term medications, compared to 46.91% in the non-hyperlipidemia group (P < 0.001). In the external validation cohort from Tongling People’s Hospital, a similar trend was observed in BMI (P < 0.001), with a higher proportion of obese individuals in the hyperlipidemia group (52.27%) compared to the non-hyperlipidemia group (29.24%). Moreover, significant differences were noted in FPG levels (P = 0.001), with the hyperlipidemia group exhibiting higher fasting plasma glucose levels.

**Table 1:** Baseline characteristics of the participants in Tongling Third People’s Hospital.

Variables	Overall (N = 368)	Non-Hyperlipidemia (N = 243)	Hyperlipidemia (N = 125)	*p*
Age, [n (%)]				0.67
18-39	83 (22.55)	57 (23.46)	26 (20.80)	
40-59	186 (50.54)	124 (51.03)	62 (49.60)	
≥60	99 (26.90)	62 (25.51)	37 (29.60)	
Gender, [n (%)]				0.12
Male	175 (47.55)	108 (44.44)	67 (53.60)	
Female	193 (52.45)	135 (55.56)	58 (46.40)	
BMI, [n (%)]				<0.001
Normal weight	78 (21.20)	78 (32.10)	0 (0.00)	
Overweight	138 (37.50)	98 (40.33)	40 (32.00)	
Obese	152 (41.30)	67 (27.57)	85 (68.00)	
Hormone disorder, [n (%)]				0.19
No	193 (52.45)	121 (49.79)	72 (57.60)	
Yes	175 (47.55)	122 (50.21)	53 (42.40)	
Diabetes history, [n (%)]				0.99
No	181 (49.18)	119 (48.97)	62 (49.60)	
Yes	187 (50.82)	124 (51.03)	63 (50.40)	
Cardiovascular disease, [n (%)]				0.75
No	197 (53.53)	132 (54.32)	65 (52.00)	
Yes	171 (46.47)	111 (45.68)	60 (48.00)	
Hypertension history, [n (%)]				0.13
No	201 (54.62)	140 (57.61)	61 (48.80)	
Yes	167 (45.38)	103 (42.39)	64 (51.20)	
Smoking, [n (%)]				0.71
No	189 (51.36)	127 (52.26)	62 (49.60)	
Yes	179 (48.64)	116 (47.74)	63 (50.40)	
Drinking, [n (%)]				0.51
No	181 (49.18)	116 (47.74)	65 (52.00)	
Yes	187 (50.82)	127 (52.26)	60 (48.00)	
Sleep disorder, [n (%)]				0.94
No	186 (50.54)	122 (50.21)	64 (51.20)	
Yes	182 (49.46)	121 (49.79)	61 (48.80)	
Bad diet, [n (%)]				0.43
No	177 (48.10)	121 (49.79)	56 (44.80)	
Yes	191 (51.90)	122 (50.21)	69 (55.20)	
Weekly physical Activity, [n (%)]				<0.001
<150 min	241 (65.49)	116 (47.74)	125 (100.00)	
≥150 min	127 (34.51)	127 (52.26)	0 (0.00)	
Long term medication, [n (%)]				<0.001
No	157 (42.66)	129 (53.09)	28 (22.40)	
Yes	211 (57.34)	114 (46.91)	97 (77.60)	
Stress, [n (%)]				0.33
No	191 (51.90)	131 (53.91)	60 (48.00)	
Yes	177 (48.10)	112 (46.09)	65 (52.00)	
Social support, [n (%)]				0.99
No	181 (49.18)	120 (49.38)	61 (48.80)	
Yes	187 (50.82)	123 (50.62)	64 (51.20)	
Life events, [n (%)]				0.12
No	196 (53.26)	137 (56.38)	59 (47.20)	
Yes	172 (46.74)	106 (43.62)	66 (52.80)	
Emotion regulation disorder, [n (%)]				<0.001
No	151 (41.03)	130 (53.50)	21 (16.80)	
Yes	217 (58.97)	113 (46.50)	104 (83.20)	
**TG (mg/dl)**	152.79 [126.78, 177.01]	149.10 [125.57, 173.60]	161.43 [127.08, 179.24]	0.27
**TC (mg/dl)**	203.34 [178.77, 224.90]	204.40 [177.99, 223.66]	199.98 [179.01, 225.93]	0.74
**LDL (mg/dl)**	132.48 [111.89, 155.78]	130.38 [111.82, 152.85]	135.98 [112.17, 158.69]	0.26
**HDL (mg/dl)**	44.29 [36.68, 53.19]	44.36 [37.22, 53.23]	43.15 [35.23, 52.71]	0.57
**FPG (mmol/L)**	102.30 [90.92, 113.71]	96.60 [87.42, 106.43]	113.71 [103.68, 122.82]	**<0.001**
**HbA1c (mmol/mol)**	5.73 [5.35, 6.15]	5.74 [5.35, 6.18]	5.69 [5.37, 6.09]	0.37
**NLR**	2.42 [1.94, 3.04]	2.42 [1.98, 3.03]	2.42 [1.90, 3.07]	0.55
**CRP (mg/L)**	5.18 [3.43, 6.86]	5.04 [3.51, 6.77]	5.46 [3.37, 7.14]	0.22

The bold values indicate p < 0.05, which means they are statistically significant.

**Table 2:** Baseline characteristics of the participants in Tongling People’s Hospital cohort. .

Variables	Overall (N = 259)	Non-Hyperlipidemia (N = 171)	Hyperlipidemia (N = 88)	*p*
Age, [n (%)]				0.71
18-39	54 (20.85)	38 (22.22)	16 (18.18)	
40-59	137 (52.90)	90 (52.63)	47 (53.41)	
≥60	68 (26.25)	43 (25.15)	25 (28.41)	
Gender, [n (%)]				0.14
Male	126 (48.65)	77 (45.03)	49 (55.68)	
Female	133 (51.35)	94 (54.97)	39 (44.32)	
BMI, [n (%)]				<0.001
Normal weight	51 (19.69)	51 (29.82)	0 (0.00)	
Overweight	112 (43.24)	70 (40.94)	42 (47.73)	
Obese	96 (37.07)	50 (29.24)	46 (52.27)	
Hormone disorder, [n (%)]				0.67
No	138 (53.28)	89 (52.05)	49 (55.68)	
Yes	121 (46.72)	82 (47.95)	39 (44.32)	
Diabetes history, [n (%)]				0.86
No	133 (51.35)	89 (52.05)	43 (47.78)	
Yes	126 (48.65)	82 (47.95)	47 (52.22)	
Cardiovascular disease, [n (%)]				0.47
No	139 (53.67)	95 (55.56)	44 (50.00)	
Yes	120 (46.33)	76 (44.44)	44 (50.00)	
Hypertension history, [n (%)]				0.32
No	148 (57.14)	102 (59.65)	46 (52.27)	
Yes	111 (42.86)	69 (40.35)	42 (47.73)	
Smoking, [n (%)]				0.80
No	125 (48.26)	84 (49.12)	41 (46.59)	
Yes	134 (51.74)	87 (50.88)	47 (53.41)	
Drinking, [n (%)]				0.43
No	128 (49.42)	81 (47.37)	47 (53.41)	
Yes	131 (50.58)	90 (52.63)	41 (46.59)	
Sleep disorder, [n (%)]				0.86
No	133 (51.35)	89 (52.05)	40 (45.45)	
Yes	126 (48.65)	82 (47.95)	48 (54.55)	
Bad diet, [n (%)]				0.61
No	122 (47.10)	83 (48.54)	39 (44.32)	
Yes	137 (52.90)	88 (51.46)	49 (55.68)	
Weekly physical Activity, [n (%)]				<0.001
<150 min	169 (65.25)	81 (47.37)	88 (100.00)	
≥150 min	90 (34.75)	90 (52.63)	0 (0.00)	
Long term medication, [n (%)]				0.66
No	133 (51.35)	90 (52.63)	43 (48.86)	
Yes	126 (48.65)	81 (47.37)	45 (51.14)	
Stress, [n (%)]				0.59
No	140 (54.05)	95 (55.56)	45 (51.14)	
Yes	119 (45.95)	76 (44.44)	43 (48.86)	
Social support, [n (%)]				0.26
No	120 (46.33)	84 (49.12)	36 (40.91)	
Yes	139 (53.67)	87 (50.88)	52 (59.09)	
Life events, [n (%)]				0.13
No	139 (53.67)	98 (57.31)	41 (46.59)	
Yes	120 (46.33)	73 (42.69)	47 (53.41)	
Emotion regulation disorder, [n (%)]				0.42
No	137 (52.90)	94 (54.97)	43 (48.86)	
Yes	122 (47.10)	77 (45.03)	45 (51.14)	
**TG (mg/dl)**	157.55 [139.80, 168.59]	154.15 [138.31, 167.19]	159.95 [145.50, 172.58]	**0.02**
**TC (mg/dl)**	203.36 [187.02, 217.85]	204.29 [186.31, 217.97]	200.97 [188.51, 217.17]	0.83
**LDL (mg/dl)**	101.64 [90.78, 113.46]	99.35 [89.85, 113.24]	103.47 [98.03, 115.32]	0.09
**HDL (mg/dl)**	48.13 [40.33, 54.96]	48.38 [40.65, 55.22]	46.99 [40.00, 53.96]	0.39
**FPG (mmol/L)**	106.91 [95.82, 114.76]	104.95 [94.23, 112.74]	110.39 [100.49, 119.75]	**0.001**
**HbA1c (mmol/mol)**	5.87 [5.48, 6.32]	5.85 [5.42, 6.33]	5.89 [5.55, 6.20]	0.32
**NLR**	1.96 [1.67, 2.40]	1.88 [1.64, 2.39]	2.05 [1.76, 2.40]	0.34
**CRP (mg/L)**	5.38 [3.93, 6.75]	5.41 [3.95, 6.75]	5.28 [3.85, 6.72]	0.89

The bold values indicate p < 0.05, which means they are statistically significant.

### Predictor selection

**Figure 2:**
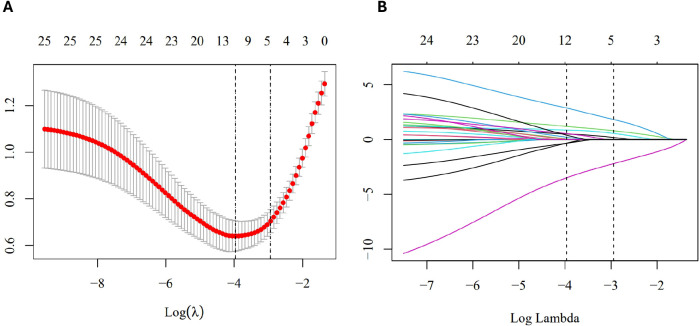
Feature selection using the LASSO regression. **(A)** Selection of the optimal penalty parameter λ via 10-fold cross-validation. The y-axis represents the Mean Squared Error (MSE), and the x-axis represents log(λ). The vertical bars indicate the standard error (SE) of the MSE. The two dashed lines represent λ_min_ and λ_1se_, respectively. **(B)** LASSO coefficient profiles of the candidate predictors. Each colored curve represents the path of the coefficient for a specific variable as the penalty increases. Variables with non-zero coefficients at the chosen λ (vertical line) were selected for the final model.

To achieve a parsimonious model and avoid overfitting, we performed Least Absolute Shrinkage and Selection Operator (LASSO) regression to screen the candidate covariates. As shown in **Figure 2A**, the optimal tuning parameter (λ) was selected using 10-fold cross-validation. Based on the λ_1se_ criterion, which favors a simpler model while maintaining predictive accuracy, six core predictors with non-zero coefficients were identified (**Figure 2B**). These predictors included BMI, weekly physical activity, long-term medication, emotion regulation disorder, CRP, and FPG.

### Multimodel integrated analysis for classification

In the multimodel integrated analysis for classification, seven machine learning models were evaluated across both the training and validation datasets, including Logistic Regression, Decision Tree, Random Forest, XGBoost, LightGBM, SVM, and ANN. As shown in **Table 3** and **Table 4**, several models demonstrated strong predictive performance; however, notable differences were observed in their performance across evaluation metrics.

**Table 3:** Performance metrics of machine learning models on the training set.

Model	AUC	Accuracy	Precision	Sensitivity	Specificity	F1 Score	Kappa	Youden’s J	PPV	NPV
Logistic	0.96	0.88	0.82	0.84	0.91	0.83	0.74	0.75	0.82	0.92
Decision Tree	0.97	0.89	0.81	0.89	0.89	0.85	0.76	0.78	0.81	0.94
Random Forest	0.97	0.89	0.92	0.77	0.96	0.84	0.77	0.74	0.92	0.89
XGBoost	0.98	0.88	0.97	0.68	0.99	0.83	0.72	0.67	0.97	0.86
LightGBM	0.97	0.89	0.86	0.79	0.94	0.83	0.75	0.73	0.86	0.89
SVM	0.96	0.87	0.75	0.92	0.84	0.83	0.72	0.76	0.75	0.95
ANN	0.96	0.88	0.82	0.83	0.91	0.82	0.73	0.74	0.82	0.91

**Table 4:** Performance metrics of machine learning models on the validation set.

Model	AUC	Accuracy	Precision	Sensitivity	Specificity	F1 Score	Kappa	Youden’s J	PPV	NPV
Logistic	0.96	0.93	0.89	0.89	0.94	0.89	0.84	0.84	0.89	0.94
Decision Tree	0.91	0.86	0.82	0.76	0.92	0.79	0.69	0.67	0.82	0.88
Random Forest	0.95	0.80	0.89	0.46	0.97	0.61	0.49	0.43	0.89	0.78
XGBoost	0.95	0.74	0.91	0.27	0.99	0.42	0.31	0.26	0.91	0.72
LightGBM	0.95	0.82	0.87	0.54	0.96	0.67	0.56	0.50	0.87	0.80
SVM	0.96	0.90	0.88	0.81	0.94	0.85	0.77	0.76	0.88	0.91
ANN	0.96	0.912	0.91	0.81	0.96	0.86	0.79	0.77	0.91	0.91

In the training set, XGBoost achieved the highest AUC (0.98), along with high precision (0.97) and specificity (0.99), indicating excellent discriminative ability. Similarly, the Decision Tree also showed strong performance, with an AUC of 0.97 and comparable sensitivity (0.89) and specificity (0.89). In the validation set, XGBoost maintained a high AUC (0.95), but its sensitivity dropped substantially to 0.27, suggesting a limited ability to correctly identify patients at high risk. In contrast, the Decision Tree model demonstrated more stable and clinically meaningful performance, achieving an AUC of 0.91 with a relatively balanced sensitivity (0.76) and specificity (0.92). Although XGBoost performed best in AUC, calibration, and decision curve analysis, its low sensitivity in the validation set makes it less suitable for clinical screening, where high-risk individuals must be reliably identified. The Decision Tree model showed a slightly lower AUC but provided comparable sensitivity and specificity, indicating better overall classification performance and robustness.

**Figure 3:**
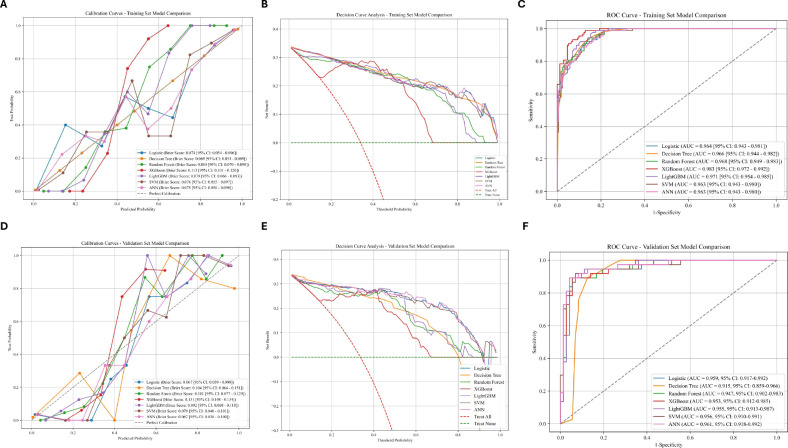
The performance and comparison of six different predictive models. **(A)** Calibration Curve for the Test Set. **(B)** Decision Curve Analysis for the test Set. **(C)** ROC Curves for the training Set. **(D)** Calibration Curve for the validation Set. **(E)** Decision Curve Analysis for the validation Set. **(F)** ROC Curves for the test Set.

The calibration curves and decision curve analysis (**Figure 3**) further supported these findings. While XGBoost showed good probability calibration and net benefit, the Decision Tree model demonstrated more consistent performance across datasets, enhancing its reliability in real-world clinical settings. The ROC curves also confirmed that multiple models achieved comparable discriminative ability, but differed in classification balance.

Considering the need for both predictive accuracy and clinical applicability, particularly in screening scenarios, the Decision Tree model was selected as the final model for subsequent external validation.

### External validation

Based on the model selection strategy described above, the Decision Tree model was chosen for external validation. The external validation of the Decision Tree model demonstrated promising predictive performance, as shown in **Table 5**. The model achieved an AUC of 0.87, accuracy of 0.79, and F1 score of 0.69, highlighting its robustness in classifying new, unseen data. Sensitivity (0.72) and specificity (0.83) further confirmed its balanced performance, with a Kappa of 0.53 and a positive predictive value (PPV) of 0.66. These results indicate that the Decision Tree model can reliably predict outcomes in an external dataset, meeting the study goal of developing a generalizable screening tool.

**Table 5:** Performance metrics of machine learning models on external validation sets.

Model	AUC	Accuracy	Precision	Sensitivity	Specificity	F1 Score	Kappa	Youden’s J	PPV	NPV
Decision Tree	0.87	0.79	0.66	0.72	0.83	0.69	0.53	0.55	0.66	0.86

**Figure 4:**
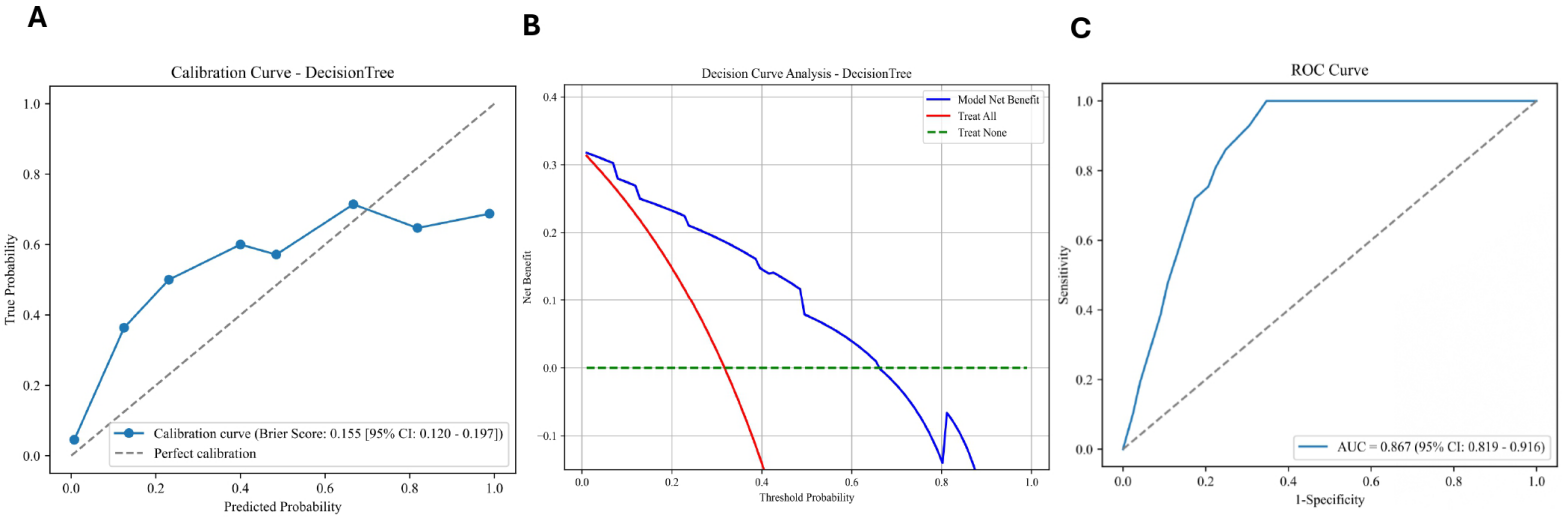
Evaluation of the decision tree model performance. **(A)** Calibration curve: The y-axis represents the true probability and the x-axis represents the predicted probability. The model demonstrated a Brier score of 0.155 (95% CI: 0.120–0.197). **(B)** Decision curve analysis (DCA): The net benefit is plotted against threshold probabilities. **(C)** Receiver operating characteristic (ROC) curve: The area under the curve (AUC) is 0.867 (95% CI: 0.819–0.916), indicating strong predictive accuracy. Note on Variable Units: Continuous predictors in the model include BMI (kg/m^2^), C-reactive protein (CRP, mg/L), and Fasting Plasma Glucose (FPG, mmol/L). Binary variables include long-term medication (Yes/No) and emotion regulation disorder (Yes/No).

**Figure 4** presents the calibration curve, decision curve analysis, and ROC curve for the Decision Tree model, further supporting its strong performance. The calibration curve suggests good agreement between predicted probabilities and actual outcomes. The decision curve analysis demonstrates a positive net benefit across a wide range of threshold probabilities, while the ROC curve confirms the model’s high discriminative power. These results underline the Decision Tree’s effective generalization to external datasets, demonstrating its practical value for early clinical identification of high-risk patients.

### Interpretability and application of the model

**Figure 5:**
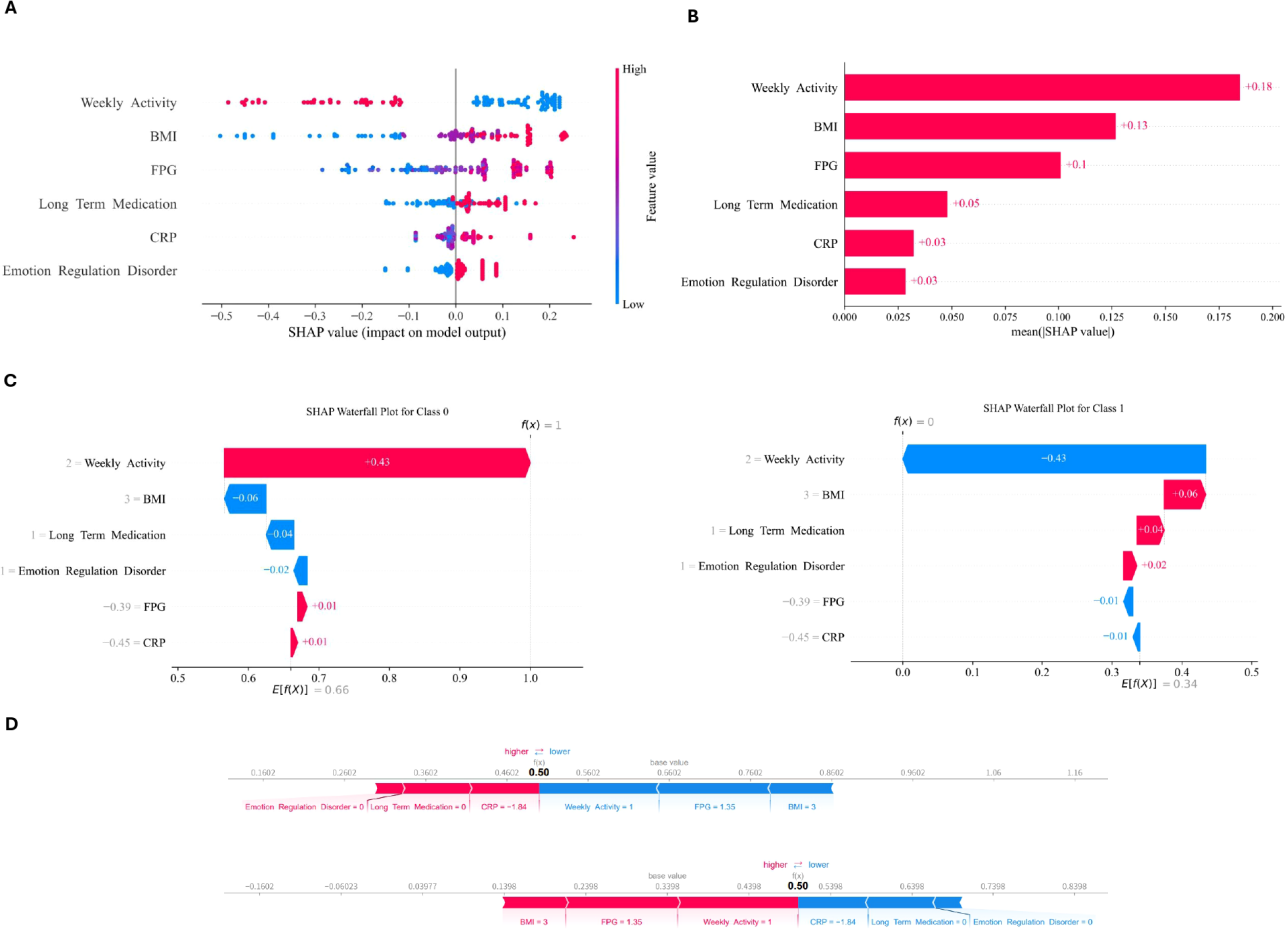
SHAP of the model. **(A)** SHAP summary showing feature contributions, with red and blue dots representing higher and lower values, respectively. **(B)** Logistic regression feature importance ranking. **(C)** Waterfall plot illustrating the combined effect of variables for a single case. **(D)** Force plot depicting individual-level risk, highlighting the balance between risk-enhancing and protective factors.

The clinical utility and interpretability of the Decision Tree model were further elucidated through SHAP visualizations as shown in **Figure 5**. In **Figure 5A**, the SHAP summary plot quantifies the global contribution of each feature, where red and blue dots represent higher and lower feature values respectively. This visualization confirms that elevated levels of clinical biomarkers, specifically BMI and CRP, are the primary drivers of hyperlipidemia risk in this population, which directly reflects the underlying biological connection between systemic inflammation and metabolic dysfunction. **Figure 5B** provides a feature importance ranking that aligns machine learning outputs with traditional clinical relevance, highlighting the critical role of metabolic indicators in the classification task. Furthermore, the individual-level interpretations provided by the waterfall plot in **Figure 5C** and the force plot in **Figure 5D** translate complex algorithmic decisions into actionable medical insights. This interpretability ensures that the model is not merely a black box but a transparent clinical application that meets the study objective of supporting personalized treatment strategies for patients with depression.

## Discussion

This study presents a novel predictive tool for secondary hyperlipidemia in patients with depression. Our final model identified six key predictors, including BMI, weekly physical activity, long-term medication use, emotion regulation disorder, CRP, and FPG, and achieved an outstanding AUC of 0.867 in the external validation cohort.

Our findings are consistent with existing evidence linking depression to metabolic and inflammatory dysregulation. The identification of BMI and FPG as dominant predictors, as demonstrated in the SHAP analysis, aligns with the work of Malmir et al. (33), who reported a strong association between elevated BMI and metabolic disorders in patients with depression. Importantly, our machine learning framework extends these findings by capturing non-linear relationships, particularly the contribution of FPG to lipid dysregulation, thereby providing a more precise risk stratification than traditional linear approaches. Similarly, the role of physical activity observed in our model supports prior evidence from Lopresti et al. (34), highlighting sedentary behavior as a modifiable risk factor in depressive populations.

Furthermore, the identification of long-term medication as an important predictor may reflect the cumulative metabolic effects of sustained pharmacological treatment. However, due to the lack of detailed classification of medication types (e.g., antidepressants or statins), the specific contributions of different drug classes could not be disentangled in the present study. Consistent with Bozdag et al. (35), our findings suggest that pharmacological treatment is closely linked to lipid alterations. Notably, our model also highlights emotion regulation disorder as a key feature, supporting theoretical frameworks proposed by Sal-Sarria et al. (36) and suggesting that psychological resilience may play a direct role in metabolic stability.

The inclusion of CRP further emphasizes the systemic and inflammatory basis of depression-related metabolic disturbances. Elevated CRP levels were associated with increased predicted risk, in agreement with findings by Kiecolt-Glaser et al. (37). Compared with previous studies that often examine metabolic or inflammatory pathways in isolation, our multimodel analysis integrates these domains, demonstrating their combined contribution to the development of secondary hyperlipidemia.

Collectively, the predictors identified in this study are well supported by prior research (38, 39), reinforcing the biological plausibility of the model. More importantly, by integrating multidimensional clinical variables, our approach provides a practical framework for early identification of high-risk individuals, directly addressing the gap highlighted in the Introduction.

This study’s innovation lies in the integration of machine learning with clinical data to predict secondary hyperlipidemia in patients with depression. The inclusion of emotion regulation disorder and CRP in our model, both less frequently emphasized in previous studies, provided new insights into the metabolic consequences of depression (40, 41). Moreover, by employing multiple machine learning algorithms, including XGBoost and Decision Tree, we ensure both robustness and high predictive accuracy. The use of LASSO regression for feature selection and external validation further enhances the model’s reliability and generalizability, making it a valuable tool for clinical practice.

Despite these strengths, several limitations should be acknowledged. First, the retrospective design and data derived from a regional healthcare system may limit generalizability to broader populations. Second, the relatively high AUC values may partly reflect the homogeneity of the study population and standardized measurement conditions. Third, the use of simplified binary variables, such as long-term medication and emotion regulation disorder, improves model interpretability but may reduce granularity. Furthermore, the observational nature of the study precludes causal inference. Future multicenter prospective studies incorporating more detailed clinical variables, including specific medication subtypes, are warranted to further validate and refine the model.

In conclusion, we developed and externally validated a machine learning model for predicting secondary hyperlipidemia in patients with depression. The Decision Tree model demonstrated robust and generalizable performance, offering a clinically interpretable tool for early risk stratification. These findings support the application of machine learning in personalized clinical management and highlight the importance of integrating metabolic, inflammatory, and psychosocial factors in risk prediction.

## Conclusion

The Decision Tree model demonstrated superior predictive performance for secondary hyperlipidemia in patients with depression, achieving an AUC of 0.97 and accuracy of 0.89 in the training set. Strong generalization was observed in the external validation set, confirming the model’s robustness. This study highlights the utility of machine learning in identifying key risk factors, offering a reliable tool for clinical decision-making and early intervention.

## Data Availability

The raw data supporting the conclusions of this article will be made available by the authors, without undue reservation.
